# Adapting a Cancer Screening Education Program for Native American Women with Disabilities

**DOI:** 10.3390/ijerph19159280

**Published:** 2022-07-29

**Authors:** Julie S. Armin, Heather J. Williamson, Andria Begay, Jennifer Etcitty, Agnes Attakai, Kim Russell, Julie A. Baldwin

**Affiliations:** 1 Department of Family and Community Medicine, College of Medicine, The University of Arizona, 655 North Alvernon Way, Tucson, AZ 85711, USA; 2 Department of Occupational Therapy, Center for Health Equity Research, Northern Arizona University, 1395 Knoles Drive, Flagstaff, AZ 86011, USA; heather.williamson@nau.edu; 3 Department of Health Sciences, Center for Health Equity Research, Northern Arizona University, 1395 Knoles Drive, Flagstaff, AZ 86011, USA; julie.baldwin@nau.edu; 4 Center for Health Equity Research, Northern Arizona University, 1395 Knoles Drive, Flagstaff, AZ 86011, USA; abb232@nau.edu (A.B.); jre227@nau.edu (J.E.); 5 Mel and Enid Zuckerman College of Public Health, The University of Arizona, 1295 N Martin Ave, Tucson, AZ 85724, USA; agnesa@arizona.edu; 6 Arizona Advisory Council on Indian Health Care, 801 E. Jefferson St., Phoenix, AZ 85034, USA; kim.russell@aacihc.az.gov

**Keywords:** Native American health, disability, cancer disparities, breast cancer, cervical cancer, cancer screenings, cancer education

## Abstract

Like other minoritized groups, people with disabilities experience lack of access to health care. People with intellectual and developmental disabilities (IDD), which are lifelong disabilities diagnosed in childhood requiring varying levels of support for completing daily activities, are less likely to receive preventive health care such as cancer screening. Furthermore, Native American women are less likely than White women to receive cancer screenings. In this qualitative research with Native American women with IDD, their caregivers, healthcare and service providers, and community leaders, we asked, “What are the influences on breast and cervical cancer screening for Native American women with IDD?” with the goal of adapting an existing cancer screening education program. Semi-structured in-depth interviews (N = 48) were audio recorded and transcribed verbatim for analysis. Two coders used a constant comparative method to code and revise the a priori codebook with subthemes and new codes. Results highlighted individual, interpersonal, and community/institutional influences on screening, emphasizing the individual effects of social inequity on this population, the importance of ableist bias in recommending cancer screenings, and opportunities to integrate traditional ways of knowing with allopathic medicine. Results of this work were used to adapt a cancer screening education program for Native American women with IDD.

## 1. Introduction

One in four adults in the U.S. report living with a disability [1]) and there is little attention paid to the intersectional experience of disability for historically marginalized populations, like Native Americans [2]. Within young adults (18–44 years) in the U.S., cognitive disabilities are most prevalent [1], including intellectual and/or developmental disabilities. Intellectual and developmental disabilities (IDD) are lifelong disabilities diagnosed in childhood that involve the individuals requiring varying levels of support for completing daily activities and can include difficulties with intellectual functioning [3]. Like other minoritized groups, people with disabilities and Native Americans experience lack of access to health care. Women with disabilities are less likely to receive cervical and breast cancer screenings compared to women without disabilities [4,5]. Additionally, Native American women do not receive recommended cancer screenings as often as other minority groups and white women [4,5].

This study is framed by ecosocial theory [6,7], which considers historical and dynamic influences, as well as discrimination, on health and lived experience. For example, people with IDD commonly live in congregate settings or with family caregivers [8], which may affect their adherence to cancer screening guidelines [9]. Furthermore, Arizona, where this study took place, is a primarily rural state and more than a third of the state’s Native American population lives in nonmetropolitan counties [10]. Rural-dwelling individuals have more limited access to cancer care and often travel long distances to access it [11,12], averaging health care trips that are nearly a third longer than trips among urban dwellers [13]. While many Native American people reside in rural tribal lands, approximately 60% of people who identify as Native American live in urban areas [14]. Both rural and urban Native American populations report using traditional healing in addition to allopathic medical care [15,16], and a number of international bodies (e.g., the World Health Organization) have acknowledged the importance of indigenous healing practices in Native American people’s lives and well-being [17]. Furthermore, despite the fact that Native Americans have a legal right to federal health care services, there may be limited access to specialty care, such as cancer care, in these systems [18]. Accordingly, this project sought to address cancer screening among Native American women IDD, a group of women who experience disparities in access to health resources.

We began with an existing breast and cervical cancer screening education program for people with IDD called *Women Be Healthy2* (*WBH2)* [19]. WBH2 has been shown to increase knowledge regarding cervical and breast cancer screenings among women with IDD [19]. Led by a community advisory board with members having expertise in cancer care, health care and/or disability resources (AA and KR are members), the team’s community engagement work found a lack of attention to disability in many cancer control programs in Arizona and a need to attend to the urban and rural contexts of Native American women with IDD. The goal of this qualitative study was to collect formative data for adapting the *WBH2* program to address the cultural context of Native American women with IDD in Arizona. In conducting in-depth interviews with Native American women with IDD, their caregivers, healthcare and service providers, and community leaders, we asked, “What are the influences on breast and cervical cancer screening for Native American women with IDD?”

Given the prevalence of disability among Native Americans in the U.S. [20], the larger implications for this culturally adapted program will be a health education resource developed in accordance with the principles of universal design [21], in which educational resources are accessible for all regardless of cognitive, sensory, or physical functioning differences. In this paper, we report the results of the qualitative data collection using concepts from the ecosocial model and discuss the implementation of these findings using social cognitive theory [22]. The adapted program is titled *My Health My Choice*.

## 2. Materials & Methods

### 2.1. Study Design and Study Participants

The consolidated criteria for reporting qualitative research (COREQ) was used to guide the reporting of methods and findings [23]. This needs assessment was intended to explore the phenomenon of breast and cervical cancer screenings among Native American women with IDD in Arizona and therefore was guided by a phenomenological approach [24]. The study was overseen by a community advisory board, who assisted with identifying potential community partner agencies to join the university-based team members in efforts to address breast and cervical cancer screening among Native American women with IDD. The community advisory board recommended that we find both a rural based partner and an urban partner to ensure the project considered both contexts related to accessing cancer screening services.

We first partnered with one community agency in this needs assessment. Our rural partner is the Hopi Cancer Support Services, a Centers for Disease Control and Prevention funded breast and cervical cancer early detection program serving Native American individuals residing in their catchment area, which is a rural region on tribal land. We did not initially find an urban community partner agency, but began recruiting urban dwelling participants while working with the community advisory board to identify this partner. After collecting some of the data in urban areas of Arizona (Phoenix, Flagstaff, and Tucson), the team was able to identify and add an urban partner, the Tucson Indian Center, a provider of health and social services to Native American people in southern Arizona. Study participants were recruited purposively based on meeting the inclusion criteria, which included either being a Native American woman with an IDD, being a caregiver to a Native American woman with an IDD, or being a community leader or provider to Native American women with IDD. Participants were recruited through email, flyers, and face-to-face interactions at local IDD and/or Native American focused events. In total, the study team members interviewed 30 rural residing individuals and 18 urban residing individuals. There were no incidences of interviewees dropping out or canceling their interview. Since the intention of the interviews was to collect information to culturally adapt a breast and cervical cancer screening education program for Native American women with IDD, the study team collected the ages of the participants who were Native American women with IDD to determine if they were at ages for which these cancer screenings were recommended. Among the Native American women with IDD, four were urban residing Native American women with IDD (age range 29–54; median age 39; age standard deviation 12.2) and eight were rural residing Native American women with IDD (age range 42–66; median age 53; age standard deviation 8.2). See Table 1 for participant demographic information.

### 2.2. Study Team, Study Setting and Data Collection

The study team was led by three doctoral-trained researchers, two who identify as White females and one who identifies as a Native American female. All three researchers have expertise in community-engaged and qualitative research. Additional team members include undergraduate and graduate students who identified as female and either White, Latinx, or Native American. For some of the rural-residing interviews with women with IDD and their caregivers, a Native American woman from a rural community with experience in research was hired to assist with recruitment and then the completion of the interviews. The study was also overseen by a Community Advisory Board who met quarterly to provide feedback on the design of the study and provided suggestions for study recruitment [25]. The study was approved by the lead university’s IRB (STUDY00000034) with a ceded review by the secondary university’s IRB. The study was also approved by all required tribally affiliated entities.

All interviewees provided informed consent prior to the interview. Informed consent materials were modified using the concepts of universal design to meet the communication skill level of the women with IDD and other participants who may have non-disclosed literacy limitations. The informed consent document used the teach-back method to ensure that study participants understood the benefits and risks of participating in the study. For all participants, the informed consent process was guided by the *Getting Involved in Research and Training Projects: A Guide for Persons with Disabilities* [26] and used simple language and imagery for individuals with low literacy levels. Consent forms were read to all individuals interested in participating in the study, and opportunities were provided for questions and answers prior to confirming consent.

After informed consent was received, semi-structured interviews were completed either in person or over the phone, depending on the person’s preferences. In most cases, only the interviewer and the interviewee were present during data collection. In three cases another person was present. This occurred as some of the women with IDD were joined by a support person for the interview (i.e., family member or paid direct support professional). There were several team members who completed interviews, including students, the lead researchers and a hired consultant, who was a Native American woman. Most of the interviewees had no previous relationships with the research team members who conducted the interviews.

The interview guides were developed by the research team. Interview guides were developed that were unique to each group including Native American women with IDD, caregivers and providers/community members. The interview guides included questions with prompts for probing regarding experiences with health care broadly, barriers and facilitators to breast and cervical cancer screenings, and preferences for learning about breast and cervical cancer screenings. Imagery was available regarding breast cancer screenings and cervical cancer screenings to assist with facilitating discussion. Materials were sent in advance to individuals who participated in phone interviews. Interviews lasted between 30 min and 1.5 h.

### 2.3. Data Analysis

Each interview was audio recorded and transcribed verbatim for analysis. Two Native American graduate students were the two coders for data analysis. An a priori codebook was initially established based on the topics discussed in the interview guides. The two coders then used a constant comparative method to review coding and update the code book as needed with codes and corresponding themes (see Appendix A for code tree). The two coders (AB and JE) provided regular updates regarding initial findings to the principal investigators (JSA and HJW) for discussion and consensus on updates to the codebook. The team determined that we had reached saturation when no new codes were added to the codebook. All data were managed using NVivo qualitative software [27].

## 3. Results

Guided by the ecosocial model, our results indicate individual, interpersonal and community/institutional influences on cancer screenings for Native American women with IDD. Many of these influences are relevant to both the urban and rural contexts, but we have noted below whether the concerns were more relevant in one context than the other. The results reported below are interviewees’ perspectives on cancer screening for themselves, their family members, the women with disabilities with whom they work, and/or the population of Native American women with disabilities.

### 3.1. Individual Influences on Cancer Screening

Providers, caregivers and women with IDD themselves described the following individual influences on cancer screenings or health care in general: financial concerns; individual attitudes about health care; fears about cancer or cancer screening; need for information about screening; general lack of health education; and beliefs about screening.

### 3.2. Financial Concerns

Financial concerns were part of the barriers to care that were highlighted among providers and caregivers. For many of the interviewees, financial burdens that come with caring for loved ones with IDD were a common barrier that resulted in individuals overlooking their health. For example, one provider commented that a young person with a disability with whom they work did not have health insurance until recently; hence, nonurgent health concerns were addressed outside of the health care system. The provider noted, “[The person’s] mom hasn’t had insurance; she doesn’t have money for that.” Once the family was able to get insurance, they visited every health care provider for checkups. (Urban Provider #1)

### 3.3. Attitudes about Health and Health Care

The behaviors, perceptions and assumptions about cancer screening can influence women’s willingness to get screened. Interviewees described the challenges of decision-making among women with IDD who might not want to have a cancer screening. Urban Provider #2 noted that a woman she supports “would really struggle” in going for a cancer screening because of the risk that she might be diagnosed with cancer. The provider commented that another medical issue nearly forced the woman with IDD to move into a group home, saying: “I could see her resisting anything that could definitely head in that direction.” (Urban Provider #2)

Providers recognized the hesitancy of the women with IDD to receive screenings and to attend appointments may be the result of a history of trauma. An urban provider described trying to have conversations with women with IDD about having gynecological exams and referenced the high rate of abuse among people with IDD [28]:

“They don’t feel comfortable talking about…did they go to the OB/GYN and that kind of stuff…I ask the support coordinator… “What are the barriers?” And she said that it’s…pretty much, that because of … a lot of high incidence of sexual abuse.”

(Urban Provider #3)

Hesitancy to get screened may also stem from encountering discrimination or bias in health care settings. One woman with IDD described her frustration with how her health care provider treats her: “She’s more concerned with my disability than anything.” (Rural Woman #1)

Providers also described feeling as if women with IDD do not get screened because they are in denial about being at risk for cancer. A rural provider noted: “But, like some of them are in denial stage…and in the long run, I see people that are being diagnosed…and all I have to say is, I wish they would just keep their appointment and get themselves checked and, you know, things will not happen to them.” (Rural Provider #1)

### 3.4. Fears about Cancer or Cancer Screening

A rural caregiver of a woman with IDD shared a perspective that reflected fear about cancer, but also a belief that individuals should know more about cancer: “You just got to know more about the cancer and, you know, how you can help, because the way I look it, cancer just takes your life. I mean, you know, there’s no turning back. You just got to live day-to-day with it....” (Rural Caregiver #1)

A rural provider highlighted negative feelings and fear regarding cancer screenings among women with disabilities. This provider also emphasized the challenge of ensuring their patients are comprehending what will be completed during the appointment:

“A lot of them with disabilities, they haven’t probably had a women’s exam for a long time, and the ones that we did do, you know, they didn’t like it…because of its being painful, and they’re not sexually active, so…I think, for them very scary and frightening and uncomfortable…so there’s got to be a way to try to make them understand what’s going to happen to them.”

(Rural Provider #2)

### 3.5. Need for Information about Cancer Screening

A rural woman with IDD described the need for information regarding cancer screenings as a beneficial approach to staying updated on her overall health. It was stated that due to their previous diagnosis of cervical cancer, they feel compelled to be informed on all the basic education on cancer screenings: “Well, I guess it doesn’t hurt to refresh, you know, your education on cancer screening. It’s been a couple of years since I had cervical cancer…So, I just need basic education.” (Rural Woman #2)

An urban woman with IDD and her caregiver described wanting more information about cancer screening. They noted that informative videos or media are hard to access due to limited internet accessibility in the group home:

“We don’t really have internet here or computers to use, so even videos are a little difficult to come by... sometimes they (health organizations) have programs where you can go online and look at things and there’s videos and descriptions, but we don’t really have access to that.”

(Urban Woman #1 and Urban Caregiver #1)

### 3.6. General Lack of Health Education for Women with IDD

An urban provider described the general lack of health education for women with IDD. The provider argued that the lack of formal and informal education has led individuals with IDD to believe the health topics (e.g., sex education) do not pertain to them.

“They may not have either the formal education about these things, or the informal education...And then sometimes they’re not included in sex education, the formal sex education that happens in schools... So, then, and you put yourself in the setting of you’re now a 50-year-old woman, and you may not have built up like a whole series of informal places that you get information...There’s a whole network of informal ways that people learn... and this goes across the board with a lot of health messaging, less accessible to people with disabilities, or they don’t see it as pertaining to them because it’s never targeted. You never see a woman in a wheelchair, for example, on a get your mammogram billboard. Right?”

(Urban Provider #5)

### 3.7. Beliefs about Cancer Screening

One woman with IDD highlighted her concern with regard to her family history with cancer, describing her interactions with the community health educators, and sharing her belief that it is important to get screened: “I had family members that passed away with cancer, and so the educators tell you, you know, it’s best to take a test…for early detection before it’s, you know, too late. Well, I like, you know, getting screened, because you never know, like you said, that out of so many that in your lifetime that you could possibly be prone to cancer, especially if it’s in your family history.” (Rural Woman #2)

### 3.8. Interpersonal Influences on Cancer Screening

Participants described the following interpersonal influences on cancer screenings or health care in general: provider and caregiver bias, the importance of communication, family and caregiver relationships, and relationship with the health care provider.

### 3.9. Provider & Caregiver Bias

Urban and rural health providers shared their feelings that provider bias may cause a barrier to screening for individuals with IDD. It was described that health providers or facilities fail to provide adequate care and attention to patients with IDD. This act often leaves patients with the feeling of being dismissed, insignificant, and overlooked. A rural provider noted:

“...sometimes I feel like in certain situations, some facilities just think that because they’re not capable of understanding these things, they don’t take the time to explain it to them, so they automatically write them off as they don’t need it or they waive their right...”

(Rural Provider #3)

Furthermore, an urban provider highlighted how health care providers tend to see the disability status first rather than the individual. The patient’s conditions or diagnoses become the focus of a patient, which limits the healthcare they receive, such as necessary preventive screening, services, and patient care.

“For people with disabilities in general, they tend not to get asked about healthcare screening and [are] more likely to be asked about stress and stuff like that. So, women particularly with disability are often assumed not to be sexually active, they never get a sexual history taken. It’s called diagnostic overshadowing…where that primary diagnosis is all the physician can see.”

(Urban Provider #5)

Support professionals commented that health care providers may not provide screenings for women with IDD due to misperceptions about risk:

“Doctors often don’t think that it’s important to do any type of cervical checks on females with a development disability. And I’ve heard medical professionals anecdotally state, “Well…they’re not sexually active, so we don’t have to worry about it…So, I think our biggest barrier, and when it comes to cervical screening, is the doctors.”

(Urban Provider #2)

Rural and urban providers commented that caregivers perpetuate these ideas about risk, thus creating barriers for tests, like cervical cancer screenings, that may also look for a commonly sexually transmitted infection (human papillomavirus, HPV), which can cause cervical cancer. A rural provider argued: “The caretakers might…feel that they don’t need to go to these appointments because they are not sexually active.” (Rural Provider #4)

### 3.10. Importance of Communication 

Providers and caregivers, in particular, highlighted the importance of communication in the health care interaction. Participants mentioned that individuals with IDD may not be able to express or communicate what they are feeling or experiencing. This requires the health care provider to effectively communicate with the patient. One urban provider described her experience:

“... if they’re having any health issues, it’s very difficult to figure out what the problem is, because either they cannot communicate….or they have some kind of speech impairment, or they just don’t know how to express...what’s happening with them.”

(Urban Provider #3)

A rural care provider highlighted that individuals with a disability may feel afraid to express what they may be going through, instead keeping it to themselves: “They’re afraid to talk about it. They might have other issues, you know, going on in their mind, but they don’t want to really tell anybody about what’s going on with them.” (Rural Caregiver #3)

Providers and health educators described utilizing various communication methods such as visual communication with pictures and diagrams to help women with IDD understand the process of screening. A rural health care provider described getting patients ready for screening:

“I usually draw out the photo...some of the girls, too, that come in for their first Pap smear, they’re already very nervous about it, so I always tell them, you know, I will draw it out, I explain what I’m doing before we do it, and then I literally like walk them through the entire exam, so they (know) what I’m doing, they feel more comfortable about it.”

(Rural Provider #7)

For an urban health care provider, patient-centered care was provided in how they communicated with their patient such as taking the time to explain in-depth the screening process and giving the patient time to feel comfortable with the screening.

“I don’t go straight to the Pap smear first thing. I may just the first time say, “I just need to look at your whole body so I’m going to sit here and have your legs go, you know, in the stirrups and I’m just going to not touch and look.” Right? Okay, six months later, I might look and then say, “Okay, now this time I’m going to touch the outside part to make sure there are no lumps and bumps,” right? And then eventually you can get to the point where sometimes you can do a Pap smear. There are patients I take care of that do not have the capacity to give consent or to understand what I’m trying to do. There are a few who, you know, their parents want them to have it. I think it’s important for them to have it. Women with disabilities are some of the highest risk for unwanted sexual assault. And so you can’t assume, you know, that somebody hasn’t been exposed to HPV even if it wouldn’t seem as if they would have had an opportunity to be exposed.”

(Urban Provider #5)

Caregivers discussed the need for cues to notify health professionals of whether a patient is non-verbal. In an interview with an urban woman with IDD and her paid caregiver, they advocated for using special tools:

“Even just the, “I communicate using” [sign] because we have some clients who are non-verbal and that would be great. Because there have been circumstances where the doctor will turn and talk to the client, but they just can’t communicate.”

(Urban Woman #1 and Urban Caregiver #1)

### 3.11. Family and Caregiver Relationships

Family and caregiver support can be essential to health care access, including screening. Caregivers are often tasked with several responsibilities such as providing transportation, interpreting, or providing general care for the woman with IDD in health care interactions.

“I have to take her in, escort her, be there with her so that the doctor can explain some things to her which I would go over, you know, tell her in [named language] what was said…to just be there with her and translate whatever it is that the doctor is getting across to her. That’s including her meds or why she can’t do this or why she can’t do that…not from me but from her doctor.”

(Rural Caregiver #3)

### 3.12. Relationship with Health Care Professionals

Women with IDD shared their thoughts about their health care providers. A rural woman with IDD commented, “I like my doctor. She’s always concerned about my disability. She’s…more one of the caring ones. It’s not where you just go in, and she does this, and so, “Okay, see you in six months.” (Rural Woman #1) An urban woman with IDD highlighted the good communication she has with her doctor, “I’m able to talk with my doctor. I’m able to explain to my doctors what’s wrong with me.” (Urban Woman #2). 

Women with IDD and caregivers acknowledged feeling more comfortable with female-identifying health care providers for cervical cancer screening. When she was asked about her last Pap test (cervical cancer screening), the same woman quoted above (urban woman #2) said that she initially had a male doctor and “...I got a little nervous. I wanted a female, but they didn’t put me with a female…. but I had two different doctors…the only one that did a Pap smear was a female doctor.” (Urban Woman #2)

A health care provider stated that they take extra steps to make their patient comfortable, respecting patient concerns and often getting on the same level as their patient:

“I really try to meet them where they’re at and not push them out of their comfort zone...[laughs]... like we’ll get them onto the exam table. Sometimes it’s hard-I’ve had a couple patients where they don’t want me to elevate the table up or they get really dizzy or anxious with that, so I have to leave the table basically pretty low to the ground, and then we still try to put their feet in the foot rests, but I kind of have to crouch on the ground sometimes, because I can’t raise the table up”

(Rural Provider #7)

### 3.13. Community or Institutional Influences on Cancer Screening

Participants described the following community or institutional influences on cancer screening: distrust of allopathic medicine, preference for traditional medicine, traditional ways of knowing, limitations to receiving community services, and accessibility of health care facilities.

### 3.14. Distrust of Allopathic Medicine

Distrust of the health professional, facility, or screening process was highlighted as a prevalent barrier for women with IDD to obtain cancer screening. Participants shared experiences with trauma, which further limits their trust in the care that may be offered to them, as expressed by an urban provider in describing a woman with whom she works.

“For her, the other thing is trauma, so she won’t get those procedures done, because she’s had the trauma sexually and physically…so to her it just relates to being victimized and, you know, you can’t justify, you know, the good behind the exam.”

(Urban Provider #4)

Additionally, when providers believe that individuals with IDD do not need cancer screenings, it further prevents these individuals from seeking or obtaining preventive care. An urban provider expressed the need for self-advocacy, acknowledging:

“... most providers don’t see that [screening is] necessary. And they [person with an IDD] can push [the health care provider], but it takes somebody to have the confidence and the know-how to push back and say, “Oh, no, you’re going to do this screening. You’re going to do this test.”

(Urban Provider #2)

### 3.15. Preference for Traditional Medicine and Ways of Knowing

Participants referred to the need to acknowledge women’s relationships with their families and their cultural context. Several rural providers described how they address women and families’ preference for traditional medicine and ways of knowing. They highlighted the need to emphasize that getting cancer does not mean that “Something bad happened or somebody did something bad and now their family’s paying for it, those kind of things.” (Rural Provider #10) They highlighted their identity as Native American and their respect of cultural knowledge to engender trust with clients. One rural provider commented that clients are, “comfortable in who we are, I guess, ‘cause we’re from their village...and we can speak and understand their language, you know, and we’re, we’re aware of our surroundings and our culture and our Native, I guess, the Native things that we do culturally.” (Rural Provider #2) Another rural provider described the strategies they use to encourage women to get cancer screenings:

“And women are the main factor in this matrilineal society even though it’s a patrilineal, you know, governing system, it’s the women that are making the decisions. It’s the women that are, you know, the ones that, you know, do the tending to the children, have the children. You know, they’re the life givers….So, if you-I notice that even here they use objects to identify with cancer like corn. ‘Okay, here’s a healthy corn. Here’s a corn that didn’t form very well and started rotting, and you know, this is what cancer can look like.’ So, they kind of, you know, weave together the good and the bad with what they’re familiar with in their environment.”

(Rural Provider #3)

### 3.16. Limitations to Receiving Community Services 

Participants described a general lack of services, with urban and rural providers, caregivers, and women highlighting lack of transportation as a major issue. For women residing in a rural area, transportation to appointments is difficult because of bad road conditions, long distances, and the lengthy time commitment to drive to appointments. A rural woman with IDD commented: “Sometimes with specialty clinics you have to go way out far like to wherever they’re held at…. the only thing that’s, you know, time consuming …is when…you have to travel far to get to your appointment.” (Rural Woman #2) A provider in a rural setting illustrated the distance needed to travel to appointments, saying: “We’re such a rural area here that, you know, if you’re at this point right here, you have a choice of going 30 min this way or an hour that way.” (Rural Provider #3)

For urban areas, there can be access to buses and transportation services like Uber and Lyft, but cost and accessibility are factors. One urban provider’s perspective highlighted the burden of cost and how it can determine if patients will attend an appointment, noting that it is a decision between “putting your money towards gas for a long trip in your truck or toward groceries that week.” (Urban Provider #5)

Furthermore, when health care facilities have limited resources or patients experience long wait times, it may dissuade women with IDD from returning for cancer screening. A rural woman with IDD commented, “One time we had to, we were there [at the clinic] most all day just to get medication. Kind of a long wait. Or that you have to go back the next day to pick it up.” (Rural Woman #3)

One rural caregiver highlighted legal concerns related to supporting a woman with IDD,, pointing to the difficulty of having caregiving responsibilities without the necessary power to carry out those responsibilities. Without the power of attorney, there can be limitations in what the health care provider can do for the patient. The caregiver stated:

“We’re not actual power of attorney of her financial or her medical, so we’ve been just doing the best we can as far as taking her to her appointments, receiving her medicine. But now because [she is on] different kinds of medicines, they’re asking for someone that’s legally medical, I guess, for her to sign these papers…we’re finding a lot of red tape right now.”

(Rural Caregiver #2)

### 3.17. Accessibility of Health Care Facilities

Health care facility accessibility is imperative to mobility for women with IDD and for caregivers who assist their patient or loved one with IDD. A rural caregiver discussed their frustration when there was limited to no proper facility accessibility: “There’s no easy way to get a screening due to her disability. In some areas it might be a closed area or just cluttered some ways to the left, [and] to the right, she’s not able to move.” (Rural Caregiver #5)

Another rural caregiver shared their dissatisfaction with inaccessible clinical spaces which require staff to transfer patients from their wheelchair to the space, commenting, “…when they’re on electric chairs, you got to get them out, transfer them, and they’re even, there’s even some members with a Hoyer lift you would have to use to transfer them” (Rural Caregiver #1).

Participants emphasized other methods for increasing accessibility, such as including the use of Indigenous languages to describe aspects of screening and providing interpreters for non-English speakers. A rural provider felt that exam room design could provide distractions to ease the worry or anxiety of the patient, recommending “making one room that’s colorful” or “making your rooms more friendly looking, bright.” (Rural Provider #8)

## 4. Discussion

Our findings address the influences on breast and cervical cancer screening for Native American women with IDD. The findings enabled the study team to hear from people who experience disability, their caregivers, and health care providers about how they understand the individual, interpersonal, and community/institutional influences on women’s receipt of breast and cervical cancer screenings.

Participants described a variety of individual influences on cancer screenings or health care in general, including financial concerns, individual attitudes about health care, fears about cancer or cancer screening, the need for information about screening, the general lack of health education, and beliefs about screening. Participant comments referenced the individual effects of inequity on the experiences of Native Americans and people with disabilities. Among people reporting disabilities, only 11.5% are uninsured in 2019 [28] but 17.7% report being uninsured at some point in the past year. People who identify as Native American are more likely to be uninsured: 32.9% were uninsured in 2019 and 37.6% were uninsured at some point in the past year. Furthermore, people with disabilities are less likely than the general population to graduate from high school. In 2018–2019, only 72% of people with disabilities graduated from high school (versus 86% in the U.S) [29,30]. Additionally, there is a lack of consideration for addressing the health literacy of adults with IDD so they can be engaged in decisions about their health care [31].

Interpersonal influences on cancer screenings or health care in general included: caregiver and provider bias, the importance of communication, family and caregiver relationships, and relationships with health care providers. In this study, participants asserted that caregivers and providers perpetuate biased ideas about women’s lack of risk for cervical cancer, in particular. They note that ideas about women with IDD as asexual may present a barrier to cervical cancer screenings. Biases about people with disabilities affect patient care more broadly. Across the United States, only 40.7% of physicians reported confidence in their ability to provide quality care to people with disabilities, and a majority (82.4%) felt that people with disabilities have worse quality of life than the general population [32]. Biases about people with disabilities can significantly affect healthcare interactions, including whether or not people with disabilities receive care. A clear example of how bias influences care was seen during the COVID-19 pandemic, during which health systems’ policies deprioritized people with disabilities for life saving medical interventions during instances of medical rationing [33]. Furthermore, as our participants noted, the role of informal or paid caregivers can be important in influencing health care. Other research has noted that strong support systems (e.g., family, friends) can influence cancer screening among people with disabilities [34].

In addition to the individual and interpersonal influences on cancer screenings, study participants also highlighted community or institutional influences on cancer screening, including distrust of allopathic medicine, preference for traditional medicine, traditional ways of knowing, limitations to receiving community services, and accessibility of health care facilities. As participants indicated, many Native American communities view allopathic medicine with apprehension due to a long history of trauma, oppression, and maltreatment [35]. Furthermore, high rates of violence and sexual assault for both women with IDD and Native American women [28,36,37] may influence the populations’ willingness to get cervical and breast cancer screenings and require providers to engage in trauma-informed care [38]. Providers who work with Native American women shared insights into bridging traditional medicine with allopathic approaches in order to engender trust but also make cancer screening relevant within women’s worldviews. 

## 5. Conclusions 

### Next Steps: Integrating Findings into the My Health My Choice Program

Formative in-depth interviews provided insight into the influences and cultural considerations necessary for adapting the original cancer screening education program, *Women Be Healthy 2*, for use with Native American women with IDD in urban and rural contexts. Using an essential elements approach [39], which identified the elements presumed to achieve the desired outcomes, we incorporated adaptations from the qualitative results reported here (Table 2) into the adapted program, *My Health My Choice*. As *My Health My Choice* is an individual intervention (vs. an intervention to address bias in the healthcare system), adaptations focused on cultivating the knowledge and skills in Native women with IDD and their caregivers to successfully advocate for their health and minimize anxiety in a health care context that may not consider their unique needs. In acknowledgement of the importance of caregivers in cancer screening adherence, the program is delivered in dyads (the Native American woman with IDD and her chosen caregiver). The COVID-19 pandemic and public health restrictions on tribal and non-tribal lands led the team to make a major change in program delivery, shifting from in-person discussions to remote delivery by teleconference or telephone. The team will then test the feasibility and acceptability and explore the effectiveness of the adapted cancer screening education program, *My Health My Choice*, with both urban and rural Native American women and their caregivers. 

## 6. Strengths and Limitations

This study reports a largely underrepresented group of people in health disparities research, Native American women with IDD. Given this study’s methodology, which enabled us to describe variations in multiple populations (e.g., urban/rural), the results of this work may not be generalizable to other groups of women with IDD who are also from underrepresented and minoritized groups. However, this work contributes to our understanding of barriers to receiving effective types of health services, in this case cancer screenings. The study also highlights the need to address multi-level issues that Native American women with IDD face when trying to access health resources. It is important to educate Native American women with IDD about cancer screenings, and in doing so, also help them plan for getting the screenings considering the personal resources available to support their health care navigation.

## Figures and Tables

**Table 1:** Participant Demographics (N = 48).

	Women with IDD		Caregivers		Providers *		Community Member/Leader		Totals
	Urban	Rural	Urban	Rural	Urban	Rural	Urban	Rural	
**Self-identified gender**									
Female	4	8	2	9	10	10	1	1	45
Male	0	0	0	1	1	1	0	0	3
**Self-identified race (may choose more than one)**									
African American	0	0	0	0	1	0	0	0	1
Latino	0	0	0	0	3	0	0	0	3
Native American	4	8	1	10	3	10	0	1	37
White	0	0	1	0	6	1	1	0	9

* Providers were health care providers or disability providers (e.g., disability program staff).

**Table 2:** Adapting the Program.

	Original Program (Woman Be Healthy 2)	Adaptations (My Health My Choice)
**What**	Knowledge about women’s health Teach skills for women to actively participate in their own health care	Emphasize traditional ways of knowing about health Highlight the importance of prevention for caregivers Activities connect advocacy skills to culturally important elements, including family and community
**How**	In-person, group discussions Interactive teaching that includes hands-on activities Topics build on each other and are delivered over time (22 sessions)	Remote delivery due to COVID-19 Dyad delivery (woman with IDD and caregiver) Six topics that build on each other but allow delivery in a shorter time
**Who**	Practitioner knows the community (e.g., women with IDD) and is known to participants	Practitioners are local health educators who are known to the community and connected to health resources Partner with local spiritual leaders and healers, in the event of a diagnosis

## Data Availability

Due to privacy and ethical concerns, the data cannot be made available.

## Appendix A. Major Code Tree

- Barriers to screening
- Benefits of cancer screening
- Continuation of care (e.g., next steps after positive screening; if screen requires diagnosis; treatment after diagnosis)
- Experience working with women with IDD
- Facilitators to screening (things that help/support screening, make it easy or easier. things that help women get screened like support services, insurance, etc.)
- Feelings about cancer
- Feelings about cancer screening
- Ideas (beliefs) about cancer
- Ideas about disability
- Knowledge of resources
- Need for information
- Patient-Centered Care
- Relationship with care provider
- Relationship with family (e.g., discussion of a person’s interactions with family)
- Relationship with health professionals (e.g., discussion of a person’s interactions with doctors, nurses, other health professionals)
- Risks of cancer screening
- Suggestions for educational program
- Suggestions for resources
- Traditional ways of knowing (e.g., understandings of cancer)
